# Supplementary material for: Laser capture microdissection in combination with mass spectrometry: Approach to characterization of tissue-specific proteomes of Eudiplozoon nipponicum (Monogenea, Polyopisthocotylea)
Source: PLoS One. 2020 Jun 17;15(6):e0231681. doi: 10.1371/journal.pone.0231681 (PMC7299319; doi:10.1371/journal.pone.0231681)
Supplement: S2 Table — (PDF) [file pone.0231681.s002.pdf]

Unique tissue-specific peptidases			
**Protein description**	**Merops ID**	**Transcriptome accession**	**Catalytic type**
**Tegument**			
Aminopeptidase	M01.003	E_nip_trans_14085_m.106065	Metallo
Leishmanolysin-like peptidase	M08.UPW	E_nip_trans_28812_m.198322	
Subfamily S8B UP	S08.UPB	E_nip_trans_69935_m.457916	Serine
**Intestine**			
Family M38 UP	M38.UPW	E_nip_trans_10356_m.66567	Metallo
Family M28 UP	M28.UPW	E_nip_trans_14053_m.105842	
Subfamily M24A UP	M24.UPA	E_nip_trans_50221_m.339700	
Subfamily M67A UP	M67.UPA	E_nip_trans_49987_m.337216	
Subfamily M67A UP	M67.UPA	E_nip_trans_67530_m.425489	
Family C95 UP	C95.UPW	E_nip_trans_02416_m.6827	Cysteine
Family C12 UP	C12.UPW	E_nip_trans_50567_m.342326	
Family C26 UP	C26.UPW	E_nip_trans_10932_m.72931	
Subfamily S9C UP	S09.UPC	E_nip_trans_11163_m.75252	Serine
Dipeptidyl peptidase	S09.UNB	E_nip_trans_38320_m.260005	
Subfamily A28B UP	A28.UPB	E_nip_trans_02587_m.8413	Aspartic

Unique tissue-specific peptidase inhibitors			
**Protein description**	**Merops ID**	**Transcriptome accession**	**Inhibitor family**
**Parenchyma**			
Family I4 UI	I04.UPW	E_nip_trans_05839_m.24152	I04
**Tegument**			
Uncharacterized protein	I21.001	E_nip_trans_16975_m.132894	I21
Family I87 UI	I87.UPW	E_nip_trans_61025_m.382655	I87
**Intestine**			
E. nipponicum KT1	I02.UPW	E_nip_trans_05461_m.23039	I02
Type I cysteine peptidase inhibitor	I25.UPA	E_nip_trans_59007_m.373320	I25
Family I29 UI	I29.UPW	E_nip_trans_06099_m.25531	I29
Family I29 UI	I29.UPW	E_nip_trans_04670_m.20209	
Uncharacterized protein	I32.UPW	E_nip_trans_07062_m.32740	I32

UP, unassigned peptidase; UI, unassigned peptidase inhibitor.
